# Monitoring the sustainable development goals through human rights accountability reviews

**DOI:** 10.2471/BLT.17.204412

**Published:** 2018-06-04

**Authors:** Judith Bueno de Mesquita, Rebekah Thomas, Camille Gauter, Alexandra Havkwist, Robert Hoddy, Ajeng Larasati, Ingrid Legrand Gjerdset, Giulia Perrone, Tasneem Sadiq, Raymond A Smith

**Affiliations:** a School of Law and Human Rights Centre, University of Essex, Wivenhoe Park, Colchester, CO4 3SQ, England.; b Department of Gender, Equity and Human Rights, World Health Organization, Geneva, Switzerland.

## Abstract

The Universal Periodic Review is a comprehensive, state-to-state peer-review mechanism of the United Nations (UN) Human Rights Council. Created in 2006, the mechanism scrutinizes the human rights record of all UN Member States, including their efforts to realize the right to health. However, the mechanism is relatively under-used in global health governance compared to treaty-based procedures, such as those overseen by the Committee on the Rights of Persons with Disabilities or the Committee on the Elimination of Discrimination against Women. We suggest that the Universal Periodic Review could be used to support the monitoring and review processes of the sustainable development goals (SDGs). The review could offer a unique perspective for other actors on how to ensure accountability for the complex and intertwined SDGs, including their commitments for health. This article provides an overview of how health-related rights have been addressed in the Universal Periodic Review process and how the review can contribute to advancing global commitments to health, including those embodied in the SDGs. We present some of the current limitations in the way health is addressed in the Universal Periodic Review. We also consider what role specialized UN agencies, such as the World Health Organization, might play during the Universal Periodic Review process and how this involvement can contribute towards the comprehensive realization of health and wellbeing for all.

## Résumé

L'Examen périodique universel est un mécanisme complet d'évaluation entre États du Conseil des droits de l'homme des Nations Unies (ONU). Créé en 2006, ce mécanisme passe en revue les réalisations de l’ensemble des États membres de l’ONU dans le domaine des droits de l’homme, et notamment leurs efforts en faveur de l'application du droit à la santé. Ce mécanisme est néanmoins relativement sous-utilisé dans la gouvernance de la santé mondiale par rapport aux procédures fondées sur des traités comme celles supervisées par le Comité des droits des personnes handicapées ou le Comité pour l'élimination de la discrimination à l'égard des femmes. Nous suggérons d'utiliser l'Examen périodique universel pour soutenir les processus de suivi et d'examen des objectifs de développement durable (ODD). L'examen pourrait offrir une perspective unique à d'autres acteurs sur la façon de garantir le principe de responsabilité pour les ODD, complexes et interdépendants, et notamment leurs engagements en matière de santé. Cet article fournit un aperçu de la façon dont les droits liés à la santé sont traités dans le cadre de l'Examen périodique universel et de la façon dont l'examen peut contribuer à faire avancer les engagements mondiaux en faveur de la santé, et notamment ceux inclus dans les ODD. Nous présentons quelques-unes des limites actuelles de l'Examen périodique universel concernant la façon dont il traite de la santé. Nous avons également étudié le rôle que peuvent jouer certaines institutions spécialisées des Nations Unies, telles que l'Organisation mondiale de la Santé, dans le cadre de l'Examen périodique universel, et en quoi ce rôle peut contribuer à l'atteinte de l'objectif de la santé et du bien-être pour tous.

## Resumen

La Revisión periódica universal es un mecanismo integral de revisión entre pares de estado a estado del Consejo de Derechos Humanos de las Naciones Unidas (ONU). Creado en 2006, el mecanismo examina el historial relativo a los derechos humanos de todos los Estados Miembros de las Naciones Unidas, incluidos sus esfuerzos por cumplir el derecho a la salud. Sin embargo, el mecanismo está relativamente infrautilizado en la gobernanza de la salud mundial en comparación con los procedimientos basados en tratados, como los supervisados por el Comité sobre los Derechos de las Personas con Discapacidad o el Comité para la Eliminación de la Discriminación contra la Mujer. Se sugiere que la Revisión periódica universal se utilice para apoyar los procesos de seguimiento y revisión de los objetivos de desarrollo sostenible (ODS). La revisión podría ofrecer una perspectiva única para otros participantes sobre cómo asegurar la responsabilidad de los complejos y vinculados ODS, incluyendo sus compromisos con la salud. Este artículo ofrece una visión general de cómo se han abordado los derechos relacionados con la salud en el proceso de la Revisión periódica universal y cómo la misma puede contribuir al avance de los compromisos mundiales con la salud, incluidos los incorporados en los ODS. Se presentan algunas de las limitaciones actuales en la forma en que se aborda la salud en la Revisión periódica universal. También se valora qué papel podrían desempeñar los organismos especializados de las Naciones Unidas, como la Organización Mundial de la Salud, durante el proceso de la Revisión periódica universal y cómo esta participación puede contribuir a la realización integral de la salud y el bienestar para todos.

## ملخص

المراجعة الدورية الشاملة هي آلية شاملة لمراجعة الحالات المناظرة بين الدول، وهي تابعة لمجلس حقوق الإنسان بالأمم المتحدة. ****أنشئت هذه الآلية في عام 2006، وهي تتولى التدقيق في سجل حقوق الإنسان لجميع الدول الأعضاء في الأمم المتحدة، بما في ذلك مراجعة الجهود الرامية في هذه الدول إلى تفعيل الحق في الصحة. ****ومع ذلك، فإن هذه الآلية قليلة الاستخدام إلى حد ما في إدارة الصحة العالمية مقارنة بالإجراءات القائمة على المعاهدات، مثل الإجراءات التي تشرف عليها اللجنة المعنية بحقوق الأشخاص ذوي الإعاقة أو اللجنة المعنية بالقضاء على التمييز ضد المرأة. ****ونحن نقترح أنه يمكن استخدام المراجعة الدورية الشاملة لدعم عمليات المراقبة والمراجعة الخاصة بأهداف التنمية المستدامة (SDG). ****يمكن أن تقدم المراجعة وجهة نظر فريدة للجهات الفاعلة الأخرى حول كيفية ضمان حدوث المساءلة عن أهداف التنمية المستدامة المعقدة والمتداخلة، بما في ذلك التزامات هذه الأهداف تجاه الصحة. ****تقدم هذه المقالة نظرة عامة عن كيفية التعامل مع الحقوق المتعلقة بالصحة في عملية المراجعة الدورية الشاملة، وكيف يمكن أن تسهم المراجعة في تعزيز الالتزامات العالمية تجاه الصحة، بما في ذلك الالتزامات الممثلة في أهداف التنمية المستدامة. ****نحن نقدم بعض المعوقات الحالية في طريقة التعامل مع الصحة في المراجعة الدورية الشاملة. ****كما نناقش أيضاً الدور الذي يمكن أن تلعبه هيئات الأمم المتحدة المتخصصة، مثل منظمة الصحة العالمية، أثناء عملية المراجعة الدورية الشاملة، وكيف يمكن لهذه المشاركة أن تساهم في تحقيق الصحة الشاملة والرفاهية للجميع.

## 摘要

普遍定期审议 (Universal Periodic Review) 是一个国与国之间的全面同级评审机制，由联合国人权理事会主持。此机制建立于 2006 年，用于定期审议联合国所有成员国的人权记录，包括他们为实现健康权所做的努力。然而，相较于基于条约的程序，例如那些受残疾人权利委员会或妇女歧视委员会监管的程序，此机制并未充分应用于全球卫生治理。我们建议将普遍定期审议用于支持可持续发展目标的监测和审议流程。当其他行为者对如何为复杂交错的可持续发展目标确认责任（包括其对健康的承诺）时，此项审议能提供一个独特视角。本文概述了普遍定期审议进程如何解决健康相关的权利问题，以及此项审议如何帮助推进实现全球对健康的承诺，包括那些体现在可持续发展目标中的承诺。我们通过普遍定期审议解决健康问题的方式展示了当前的一些局限。我们也考虑到联合国专门机构（如世界卫生组织）在普遍定期审议流程中可能发挥的作用，以及此类参与将如何为全面实现人类的健康幸福做出贡献。

## Резюме

Универсальный периодический обзор представляет собой механизм всеобъемлющего межгосударственного коллегиального обзора Совета по правам человека Организации Объединенных Наций (ООН). Этот механизм, созданный в 2006 году, анализирует данные о соблюдении прав человека во всех государствах-членах ООН, включая их усилия по реализации прав человека на здоровье. Однако этот механизм в системе руководства глобальным здравоохранением используется в недостаточной мере по сравнению с такими договорными процедурами, как процедуры, контролируемые Комитетом по правам инвалидов или Комитетом по ликвидации дискриминации в отношении женщин. Авторы считают, что универсальный периодический обзор можно использовать для поддержки процессов мониторинга и обзора целей в области устойчивого развития (ЦУР). Этот обзор может предложить уникальную перспективу для других участников в отношении того, как обеспечить подотчетность комплексных и тесно связанных ЦУР, включая их обязательства в отношении охраны здоровья. В данной статье представлен процесс рассмотрения универсальным периодическим обзором вопросов соблюдения прав в области охраны здоровья, а также как этот обзор может способствовать продвижению глобальных обязательств в отношении охраны здоровья, в том числе тех, которые включены в ЦУР. Авторы представляют некоторые из существующих ограничений в отношении того, каким образом в универсальном периодическом обзоре рассматривается вопрос охраны здоровья. Также рассматривается вопрос о том, какую роль могут сыграть специализированные учреждения ООН, такие как Всемирная организация здравоохранения, в процессе универсального периодического обзора и как это участие может способствовать всесторонней реализации прав в области охраны здоровья и благополучия всего человечества.

## Introduction

The Universal Periodic Review is a state-led, peer-review mechanism of the inter-governmental Human Rights Council that reviews United Nations (UN) Member States’ fulfilment of international human rights standards.^1^ The review complements, rather than duplicates, existing UN human rights review processes, such as the UN human right treaty bodies, which are committees of independent experts that review States parties’ compliance with international human rights treaties.^2^^,^^3^

The Universal Periodic Review is one of the most widely endorsed international human rights accountability tools.^4^ It supports the promotion and protection of human rights and assists states in building their capacity to protect and promote human rights through technical assistance and best practice sharing.^5^

Created in 2006, the review assesses, on a rotating basis, each UN Member State’s human rights record, including the right to health. In 2017, the Universal Periodic Review entered into its third cycle having completed two full reporting rounds in 2008–2012 and 2012–2016. The review draws from three sources of information: (i) a national report provided by the state under review; (ii) a compilation report of UN information on the state under review prepared by the Office of the UN High Commissioner for Human Rights, including information from UN human rights mechanisms and other official UN documentation, which can be provided by UN agencies and country teams; and (iii) a stakeholders report, which summarizes information provided by other actors, notably civil society and national human rights institutions. Following a discussion with representatives of the state under review and representatives of all 47 members of the Human Rights Council, the state under review is issued with recommendations. The state indicates which recommendations it supports, which signals a commitment to implementation. The state “notes” the recommendations it does not support.

The Universal Periodic Review process has several shortcomings, including the risk of state-to-state complicity in how recommendations are framed,^4^ a general lack of specificity in structure and delivery of some recommendations^6^ and low levels of implementation.^7^ Despite these shortcomings, the process has been widely seen as a success and has taken a central role in global human rights protection.^4^

Indeed, the Universal Periodic Review has some unique features that sets the review apart from other human rights mechanisms. In contrast to UN human rights treaty bodies, which focus on specific rights or groups, such as people with disabilities or women, the Universal Periodic Review is comprehensive, reviewing all UN member states and all human rights standards, irrespective of a whether or not a state has ratified a particular treaty. States report on time to the Universal Periodic Review, while the periodic country reports submitted to the treaty bodies are often overdue and in some cases not submitted at all.^8^ In addition to the formal legal standards of international human rights law, the review also considers voluntary pledges and commitments made by states. In discussions among the working group that oversaw the establishment of the Universal Periodic Review in 2006,^9^ some Member States suggested that these commitments might include those arising from various world conferences and summits, such as the Vienna Declaration and Programme of Action. While the most recent guidelines for submission to the Universal Periodic Review does not make this explicit,^10^ it paves the way for the possibility that the Universal Periodic Review could be used to monitor the sustainable development goals (SDGs). Unlike some other human rights monitoring mechanisms, the Universal Periodic Review process was created to be cooperative, rather than confrontational, emphasizing the role of organizations like WHO to constructively help states to meet their human rights obligations. In this article, we provide an overview of how health-related rights have been addressed in the Universal Periodic Review process and how the review can contribute to advancing global commitments to health. We also discuss what role specialized UN agencies, such as the World Health Organization (WHO), might play during this process and how this involvement can contribute towards the comprehensive realization of health and wellbeing for all.

## Consideration of health-related rights

Health is recognized as a human right under international human rights law, including the Universal Declaration on Human Rights (1948) and the International Covenant on Economic, Social and Cultural Rights and is also a central commitment in the SDGs.^11^ Furthermore, health is a prominent theme in the Universal Periodic Review recommendations made to states.

**Fig. 1:**
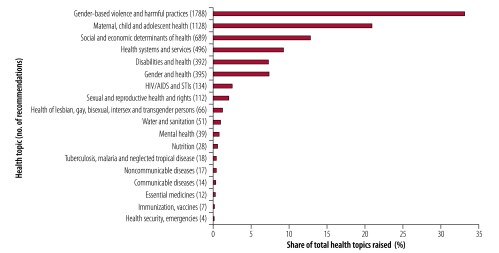
Distribution of health-related topics in the recommendations from the first cycle of the Universal Periodic Review, 2008–2012

AIDS: acquired immunodeficiency syndrome; HIV: human immunodeficiency virus; LGBTI: lesbian, gay, bisexual, transgender and intersex; STI: sexual transmitted infections.

Notes: In total, we assessed 3862 health-related recommendations. A recommendation could mention more than one topic. In total, 5390 health-related topics in all member states were mentioned in these recommendations.

To assess the presence of health-related recommendations, we analysed all the recommendations made to Member States during the first cycle of the Universal Periodic Review and counted the numbers of paragraphs relevant to health, as well as on each health related topic. By using a relatively broad interpretation of health, including many of the social and economic determinants of health (Fig. 1), we found that 3862 (22%) of the 17 638 paragraphs of recommendations were health-related.^12^ A sample review of recommendations made to a geographically diverse selection of eight countries in the second cycle of the Universal Periodic Review (2012–2016) suggests that health recommendations were more frequently made during this cycle, both in terms of absolute numbers and as a proportion of all recommendations. In these countries, health-related recommendations increased from 203 to 432 recommendations and from 20% to 26% of total recommendation between the two cycles. We also examined the stratification of recommendations by WHO Regions and found broadly similar patterns in both number and proportion of health recommendations across regions.^12^

Within the health-related recommendations from the first cycle, gender-based violence was the most frequently mentioned topic (33%; 1788 of 5390 mentions of health-related topics (Fig. 1)). Maternal and child health was the second most frequent topic of total health-related issues raised (Fig. 1) (21%; 1128 of 5390 mentions of health-related topics recommendations, however with few adolescent health recommendations), followed by social and economic determinants of health (13%; 689 of 5390 mentions of health-related topics) and health systems and services (9%; 496 of 5390 mentions of health-related topics). All of these health-related topics are prominent among the targets of the SDGs, notably the SDG 3 targets to achieve universal health coverage and reduce maternal, newborn and child mortality, and the SDG 5.2 target to eliminate all forms of violence against women and children. Recommendations often coupled health with other human rights issues, such as education or gender equality, highlighting the indivisibility of health with the enjoyment of other human rights. This holistic and synergistic approach also underpins *Transforming our world: the 2030 agenda for sustainable development*.^11^

The commitment of the 2030 agenda “leaving no one behind” which resonates with the human rights principles of equality and non-discrimination, is also apparent in the recommendations, especially on health issues affecting marginalized groups, and particularly women and children, and to some extent rural populations, people in poverty and migrants. Yet the right to health of other groups, such as adolescents, people with disabilities and minorities (with some exceptions, notably Roma populations in Europe) received much less attention.

Not all health topics received the same degree of scrutiny in the recommendations. Several health topics, including mental health and noncommunicable diseases, that are increasingly prominent and encompassed by the SDGs were comparatively neglected in recommendations. Some social determinants of health, notably water, sanitation and nutrition, as well as to access to medicines were rarely mentioned in the recommendations. Perhaps less surprisingly, given the peer-review nature of the mechanism, politically sensitive or contentious health issues, such as safe abortion, were also rarely mentioned. These findings are supported by a compilation of Universal Periodic Review recommendations relevant to the SDGs, showing a dominance of health recommendations related to universal health coverage, communicable diseases and maternal and child health.^13^

The recommendations from the first cycle largely reflect the narrower global health and well-being agenda at the time the Universal Periodic Review was undertaken. Maternal, child and reproductive health, which were addressed in the millennium development goals (MDGs), all featured in the recommendations made to states. We anticipate this focus could change as the broader vision of health reflected in the SDGs expands the way that the right to health is understood and addressed. Nonetheless, the key MDG issues of human immunodeficiency virus and acquired immunodeficiency syndrome issues (HIV/AIDS) and water and sanitation were neglected in first cycle recommendations. This suggests that monitoring the evolution of health-related recommendations throughout the third cycle, and engaging with reporting and reviewing states on the right to health, will be important. Such monitoring could ensure that stakeholders adopt a broad understanding of the right to health and its relationship to other underlying determinants as well as its link to broader peace and security goals.

## SDG accountability

Widely lauded for its universal and comprehensive reach, the 2030 agenda was born from a recognition that global development challenges are overlapping and interconnected. The agenda also seeks to address growing inequalities within and between countries, noting that these can only be bridged by tackling the complex social and structural barriers facing those left behind by development progress.^11^ These recognitions reflect the understanding of human rights as indivisible, interrelated and interconnected, and the principles of equality and non-discrimination, which underpin international human rights. Indeed, the 2030 agenda is explicitly grounded in international human rights law.

The 2030 agenda is committed to a process of voluntary follow-up and review, at the national, regional and international levels. At the international level, formal follow-up and review arrangements have centred on the adoption of an official set of indicators to monitor progress towards the goals and targets. The High Level Political Forum under the auspices of the UN General Assembly has begun assessing progress through thematic and voluntary country reviews. Yet commentators have referred to the importance of accountability for the SDGs,^14^^,^^15^ and stakeholders have recognized that the Universal Periodic Review could play a valuable role in several ways.^16^^,^^17^

First, the Universal Periodic Review provides an opportunity to assess how the SDGs are contributing to the realization of human rights, including the right to health and how human rights contribute to the SDGs.^18^ The SDGs have already been explicitly discussed in some reports submitted under the Universal Periodic Review, during the review process and in recommendations issued to states under review. The Human Rights Council has signalled its willingness to developing this practice. Using the 2030 agenda together with international human rights instruments as a framework of reference for reporting, reviewing and recommendations, should help the Universal Periodic Review broaden its focus to address health and its determinants in a more even manner.

Second, the recommendations of the Universal Periodic Review provide valuable insights into some of the health issues and population groups that need attention in every country if progress is to be made towards attainment of the SDGs. Moreover, the review provides information that can ensure that this progress is grounded in human rights, which is a commitment of the 2030 agenda. States can thus integrate recommendations received under the Universal Periodic Review, as well as from other human rights review processes, in the development, implementation and review of their efforts to achieve the SDGs. Even where recommendations are not explicitly framed in terms of the SDGs, a database developed by the Universal Rights Group allows the user to identify specific Universal Periodic Review recommendations to each state that are relevant to its SDG efforts.^13^

Third, notwithstanding the shortcomings noted above, the Universal Periodic Review offers a unique insight into the challenges that will be faced by states in reporting on the SDG agenda, which, like international human rights law, has a comprehensive scope and complex and intersecting targets. Review recommendations are at times very sweeping and broad and many recommendations cover multiple issues. Sometimes health is addressed among a broader range of issues relating to other rights, such as food, water and education. Even within the health-focused recommendations, distinct health issues are clustered together, making it difficult to separate out which, if any, health issues are to be prioritized. Our study findings suggest that this overlap appears to have implications for how states can measure and report on implementation of recommendations.^12^ These experiences of reporting under the Universal Periodic Review can and should inform the still-evolving reporting, review and recommendations and follow-up arrangements for the SDGs.

These contributions of the Universal Periodic Review are important because the formal international SDG monitoring arrangements are falling short on human rights.^19^ For example, human rights considerations are not consistently reflected in the SDG indicators and thus may be overlooked.^20^ While the High Level Political Forum provides for a process of voluntary state review, human rights have been inconsistently and inadequately addressed in this process to date.^21^ In this respect, the Human Rights Council, has already signalled its commitment to supporting the High Level Political Forum.^18^

However, if the Universal Periodic Review is to be truly valuable, it must more consistently pay attention to a broader spectrum of health issues and give even greater attention to a range of vulnerable groups, rather than clustering recommendations in an uneven way. Furthermore, the quality of recommendations can be improved and states must give more attention to implementation.

## Monitoring progress

To determine the effectiveness of the Universal Periodic Review mechanism, researchers have developed various approaches to measure the extent to which recommendations have been implemented and have triggered change in reality.^7^ Using the mid-term implementation assessments conducted by UPR Info, a nongovernmental organization, we were able to assess 156 of the 203 health-related recommendations made to eight countries during the first cycle. We found that 32 (21%) health-related recommendations were considered to be fully implemented and 64 (41%) partially implemented after two and a half years.^12^ A separate study by UPR Info also found that right to health recommendations had a comparatively high level of implementation.^7^ The study reports that 64% of right to health recommendations were fully or partially implemented, compared with an average of 48% among all recommendations. Recommendations on HIV/AIDS issues were highly implemented (78% fully or partially implemented).^7^

Cognisant of the challenge to ensure implementation of the many recommendations issued to states under the Universal Periodic Review, state reviews should in the third universal periodic review cycle pay adequate attention to progress on recommendations issued in earlier cycles. Reviews should also facilitate more effective follow-up procedures at the national level.

Lesson learnt from those recommendations that report the highest rates of implementation might be useful information that could facilitate the role of Universal Periodic Review in strengthening progress towards and accountability for the SDGs. For example, substantively narrower and more specific wording could help by making recommendations more targeted to specific problems and also enable clearer accountability for non-compliance.^7^ Yet broader language in recommendations may, in some cases, allow for more inclusive political dialogue, particularly around sensitive issues. The comparative strengths of targeted versus more general recommendations merits further research and evaluation. WHO’s normative standards on health might help navigate between the political sensitivities of some of these topics and provide a clear and evidence-based guidance on how to ensure and improve health outcomes and respect for human rights.

## Multilateral organizations

The Universal Periodic Review is an inclusive process providing multiple entry points for diverse stakeholders, including UN agencies, to contribute across the review and reporting cycle. However, historically, only a few multilateral organizations have routinely engaged in this procedure.

The UN Secretary General commented on this gap in his 2017 report *Strengthening of the United Nations action in the field of human rights through the promotion of international cooperation and the importance of non-selectivity, impartiality and objectivity*.^22^ He noted the opportunity of the 2030 agenda as “a catalyst for national implementation efforts and key entry point for the constructive engagement of the United Nations with Member States for the promotion and protection of human rights.” Furthermore, in the Report of the Secretary-General on the work of the Organization, he urged UN programmes “to strengthen the relevance, precision and impact of the [Human Rights Council] including by providing better support to Member States in implementation, stronger collaboration with United Nations country teams and…to link the Universal Periodic Review to the implementation of the [SDGs].”^23^

The prominence of health in the first two Universal Periodic Review cycles offers opportunities for organizations engaged in global public health to support implementation of these recommendations in-country and to ensure sustained attention to them at global level. In this context, we argue that WHO could play a much more influential role in the Universal Periodic Review process, through increased collaborative data sharing and to provide more technical assistance.

As a trusted source of knowledge and data, WHO country offices could contribute data on key health challenges into the Universal Periodic Review process, through the UN compilation report. This contribution would help to highlight gaps, challenges and best practices. Furthermore, WHO would provide states with evidence-based technical normative guidance to support the effective implementation of recommendations designed to help states meet their obligations under the right to health. Such involvement would align with WHO’s role to promote the use of data for global, regional and national accountability. Furthermore, WHO’s 13th Global Programme of Work states “Health is fundamental to the SDGs and, in an interconnected world, WHO’s role in providing global public goods that help to ensure health for all people within and across national boundaries has never been more relevant. The Organization’s powerful voice for health and human rights is indispensable to ensure that no-one is left behind.”^24^ This statement provides further support of the alignment of WHO’s work to the Universal Periodic Review.

## Conclusion

The Universal Periodic Review process offers an opportunity to identify and expose important health-related human rights issues and to generate action and attention in countries. Recent debates among human rights advocates on how to improve the Universal Periodic Review have focused on ensuring that previous accepted recommendations are implemented by the states before they enter the third cycle. However, to date, few people have examined how well the process achieves its ambitious goal of assessing human rights in a comprehensive, interrelated and holistic way. This kind of a comprehensive approach is required under the 2030 agenda, which recognizes that health is linked to the other 16 goals, and is dependent on the their achievements.

Our research shows that the Universal Periodic Review can and does address health from a human rights perspective. However, the current Universal Periodic Review reporting process is skewed, with attention diverted towards a narrow scope of issues. Organizations, such as WHO, that are uniquely positioned to support and provide crucial insights into the process with regard to health rights, could be doing more to ensure that these reviews are as comprehensive as possible.
